# Molecular Docking Studies and Anti−Snake Venom Metalloproteinase Activity of Thai Mango Seed Kernel Extract

**DOI:** 10.3390/molecules14093198

**Published:** 2009-08-27

**Authors:** Pimolpan Pithayanukul, Jiraporn Leanpolchareanchai, Patchreenart Saparpakorn

**Affiliations:** 1 Department of Pharmacy, Faculty of Pharmacy, Mahidol University, Bangkok 10400, Thailand; E-mail: ry_110@yahoo.com (J.L.); 2 Department of Chemistry, Faculty of Science, Kasetsart University, Bangkok 10900, Thailand; E-mail: patchareenart_s@yahoo.com (P.S.)

**Keywords:** anti−snake venom metalloproteinase activity, *Calloselasma rhodostoma*, *Mangifera indica* L., molecular docking study, *Naja naja kaouthia*, pentagalloylglucopyranose

## Abstract

Snakebite envenomations cause severe local tissue necrosis and the venom metalloproteinases are thought to be the key toxins involved. In this study, the ethanolic extract from seed kernels of Thai mango (*Mangifera* *indica* L. cv. ‘Fahlun’) (Anacardiaceae) and its major phenolic principle (pentagalloylglucopyranose) exhibited potent and dose−dependent inhibitory effects on the caseinolytic and fibrinogenolytic activities of Malayan pit viper and Thai cobra venoms in *in vitro* tests. Molecular docking studies revealed that the binding orientations of the phenolic principles were in the binding pockets of snake venom metalloproteinases (SVMPs). The phenolic principles could form hydrogen bonds with the three histidine residues in the conserved zinc−binding motif and could chelate the Zn^2+^ atom of the SVMPs, which could potentially result in inhibition of the venom enzymatic activities and thereby inhibit tissue necrosis.

## 1. Introduction

Snakebite envenomations constitute a relevant public health hazard in remote areas of Thailand since the economic activities in these areas are mainly agricultural. In Thailand, *Naja naja kaouthia* Lesson (NK) (Thai cobra, Elapidae) causes a high mortality rate due to snake envenomation [1] and *Calloselasma rhodostoma* Kuhl (CR) (Malayan pit viper, Viperidae) causes the greatest incidence of envenomation in the country [2]. Among the many venomous snakes found in Thailand, CR and NK venoms cause the most severe local tissue damage on the bite site.

Snake venoms are complex mixtures and are comprised mainly of proteins and peptides possessing a variety of biological activities. Snake venom metalloproteinases (SVMPs) are the only venom components that possess hemorrhagic activity and are both direct [3] and indirect [4] mediators of local tissue damage, such as hemorrhage, edema, myonecrosis, dermonecrosis and inflammation following envenoming. Most of the venom metalloproteinases are fibrinogenolytic enzymes, cleaving preferentially the Aα−chain and slowly the Bβ−chain of fibrinogen [5]. Substrate specificities of metalloproteinases have been assayed with casein, insulin B peptides and intermolecularly quenched fluorogenic peptide substrates [6]. SVMPs are responsible for degrading extracellular matrix (ECM) proteins, such as collagen IV, laminin, fibronectin, and the proteoglycan perlecan, which provide a supporting scaffold for endothelial cells of blood vessels [7]. These enzymatic toxins cause localized hemorrhage, either through damage to endothelial cells [8] or through gaps produced between endothelial cells as a result of damage to the basement membrane of blood vessels [5]. Hemorrhage produced by SVMPs can subsequently lead to edema, shock, tissue necrosis, and reduced ability to regenerate muscle tissue [3]. Additionally, leakage of blood from affected vessels also helps spread other venom toxins to their target tissues. Therefore, it can be hypothesized that the inhibition of these enzymes may result in a significant overall reduction in local tissue damage following envenomation [9].

Hemorrhagic and fibrinogenolytic metalloproteinases have mainly been purified from the crotalid and viperid venoms, but recently, some interesting homologues have also been isolated from elapid venom. SVMPs are a subgroup of the reprolysins and have two characteristic features: the conserved zinc−binding motif HEbxHxbGbxHD (b: bulky hydrophobic residue; x: any residue) that contains the catalytic glutamate residue, and the so−called Met−turn, a methionine−containing 1,4−β−turn that serves as a base for the three active histidine residues [10,11]. Depending on the domain composition, SVMPs can be classified as belonging to one of four classes: P−I, the smallest metalloproteinases, possess molecular masses of 20−30 kDa and contain only the protease domain. Rhodostoxin (25 kDa), a major SVMP from *Calloselasma rhodostoma* is an example of this group. P−II, the medium−sized enzymes with molecular masses of 30−50 kDa, have additionally a disintegrin−like domain. P−III are the most potent hemorrhagic toxins with a molecular masses of 50−80 kDa and a third domain (cysteine−rich domain). Kaouthiagin (51 kDa), isolated SVMP from *Naja kaouthia* is an example of this group. P−IV are enzymes that comprise all the aforementioned domains and are linked by a disulfide bridge to a type−C lectin subunit [6].

Intravenous administration of equine or ovine−derived antivenins constitutes the mainstay in treating snakebite envenomations. However, due to the rapid development of local tissue damage [12], and since antivenins are available mainly in health facilities, these products are usually administered when local effects have developed to some extent. Therefore, their neutralization is achieved only partially [13]. Consequently, there is a need to develop alternative approaches that may be applied in the field, complementing antivenom therapy in the neutralization of venom−induced local effects.

Tannins from plants have been shown to interact with enzymes from snake venoms and act as an antidote [14,15,16,17,18,19,20]. The ethanolic extract of Thai mango seed kernels (MSKE) (*Mangifera indica* L. cv. ‘Fahlun’, Anacardiaceae) has a relatively high phenolic content and is composed of 1,2,3,4,6−penta−*O*−galloyl−β−D−glucopyranose (PGG) (61.28%), methyl gallate (MG) (0.68%) and gallic acid (GA) (0.44%) [21]. We have recently demonstrated that the extract and its phenolic principles exhibited potent anti−enzymatic activities on phospholipase A_2_, hyaluronidase, and L−amino acid oxidase in *in vitro* tests and exhibited anti−hemorrhagic and anti−dermonecrotic activities against CR and NK venoms in *in vivo* tests [18]. The aim of this study was to further investigate the inhibitory effect of MSKE and its isolated phenolic principles on the *in vitro* proteolytic activity of (metallo)proteinases from CR and NK venoms using casein and fibrinogen as substrates. We also performed molecular docking studies using the Gold version 4.0 program with the aim of explaining the differences in activity of the plant polyphenols isolated from MSKE. Understanding how the tannin principles in the extract interact with zinc−metalloproteinases (rhodostoxin and kaouthiagin from CR and NK venoms, respectively) may explain how they inhibit the toxins.

## 2. Results and Discussion

### 2.1. In vitro test for the inhibition of caseinolytic activity

**Figure 1:**
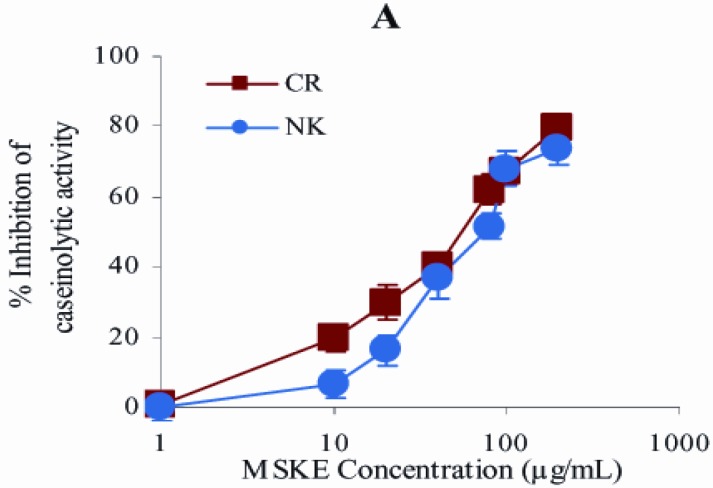

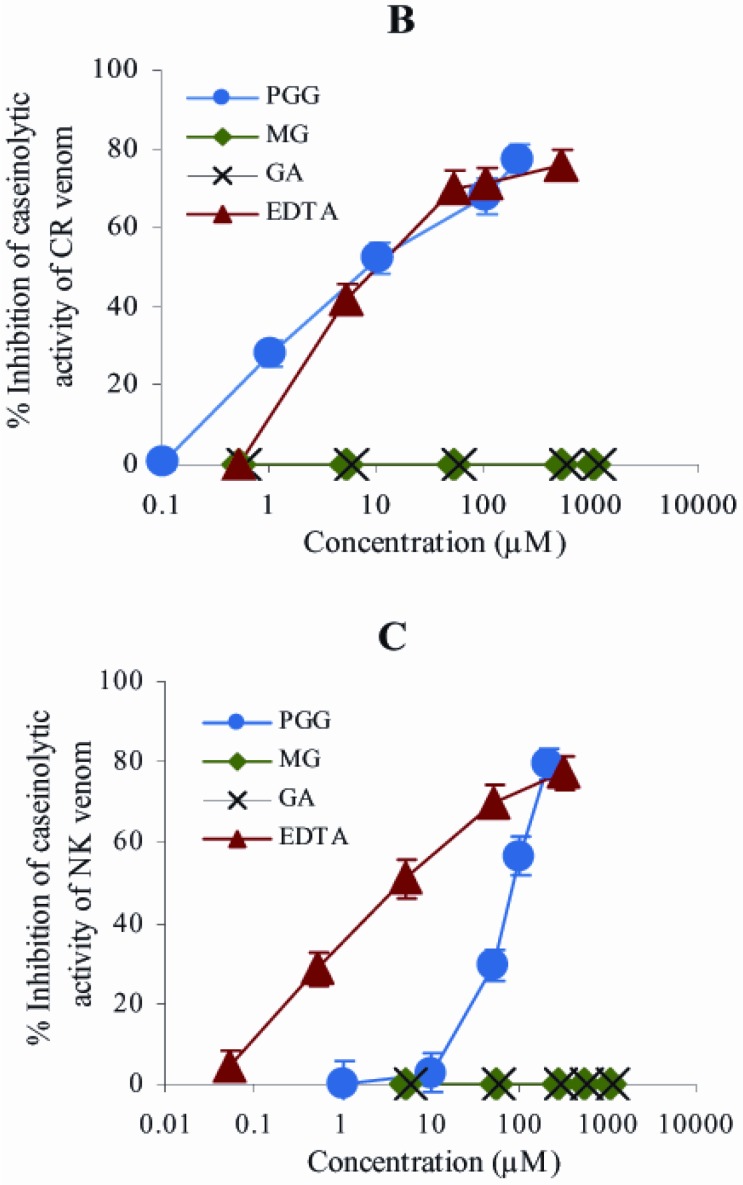
Semi−log plots of *in vitro* inhibition of caseinolytic activity of CR (50 μg) and NK (500 μg) venoms by MSKE (A), EDTA and the phenolic principles of MSKE (B, C). Values are mean ± S.E.M. of at least duplicate experiments.

From the *in vitro* test, the initial caseinolytic doses of CR and NK venoms obtained from the plot between venom doses and their caseinolytic activity were 50 and 500 µg, respectively. Figure 1 demonstrates that MSKE, its major phenolic principle (PGG) and EDTA inhibited the caseinolytic activity of both venoms in a concentration−dependent manner. The effective concentrations (EC_50_s) of the extract at which 50% of the caseinolytic activity of CR and NK venoms was inhibited were 58.2 ± 2.4 and 75.8 ± 2.1 μg/mL, respectively, (Figure 1A). 

The order of potency of PGG and EDTA as judged by the EC_50_ was in the following order: PGG (9.5 ± 2.2 μM) > EDTA (19.0 ± 1.9 μM) for CR venom (Figure 1B), and EDTA (5.0 ± 2.7 μM) > PGG (91.5 ± 2.6 μM) for NK venom (Figure 1C). Neither GA nor MG inhibited the caseinolytic activity of both venoms.

### 2.2. In vitro test for the inhibition of fibrinogenolytic activity

**Figure 2:**
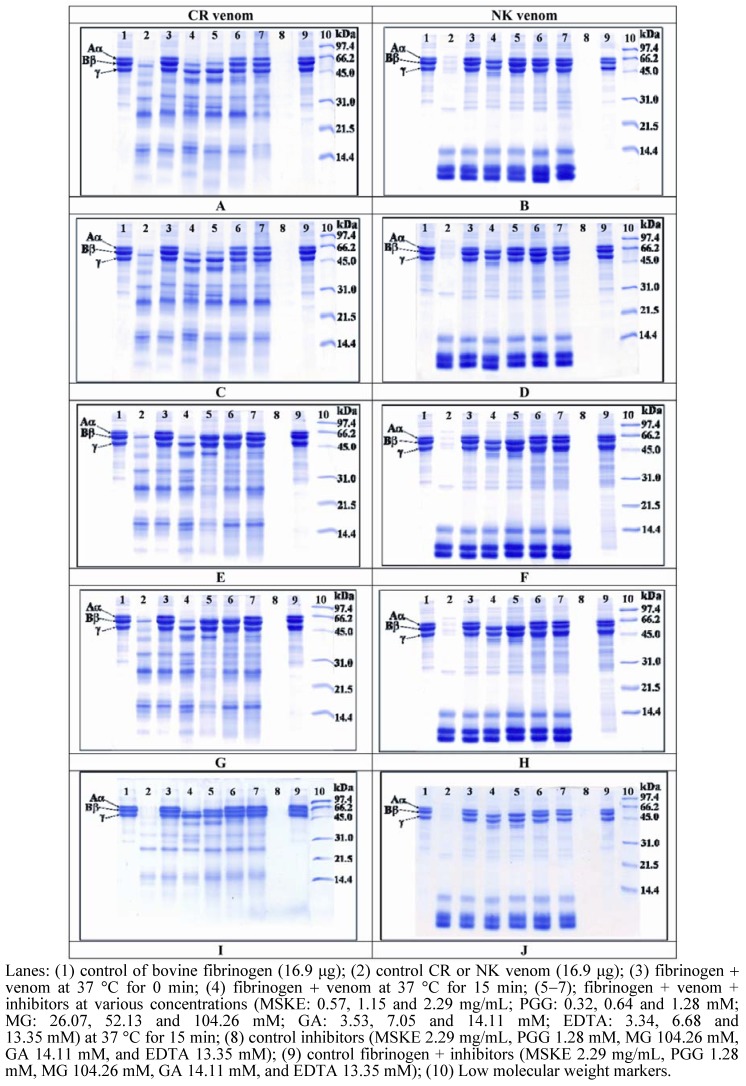
*In vitro* inhibition of fibrinogenolytic activity of CR (left) and NK (right) venoms by MSKE (A and B), PGG (C and D), MG (E and F), GA (G and H), and EDTA (I and J).

Figure 2 demonstrates that CR venom was able to degrade both Aα−and Bβ−chains of bovine fibrinogen, more preferentially the Aα−chain whereas NK venom was able to degrade only the Aα−chain of bovine fibrinogen. MSKE and its phenolic principles (GA, MG and PGG) and EDTA inhibited the fibrinogenolytic activity of both CR and NK venoms in a concentration−dependent manner. The concentrations of MSKE at which the fibrinogenolytic activity of CR and NK venoms was inhibited completely were 1.15 and 0.57 mg/mL, respectively, (Figure 2A and Figure 2B). The concentrations of EDTA and the phenolic principles of MSKE that completely inhibited this activity of both venoms were in the following order: PGG (0.64 mM) > EDTA (6.68 mM) > GA (14.11 mM) > MG (104.26 mM) for CR venom and PGG (0.32 mM) > EDTA (6.68 mM) > GA (7.05 mM) > MG (52.13 mM) for NK venom.

The most likely mechanism for the inhibition of caseinolytic and fibrinogenolytic activities by EDTA may be due to its chelating properties, which may result in chelation with the divalent metal ions (especially Zn^2+^ and Ca^2+^) of these enzymes, causing inhibition. MSKE and its phenolic principles may also have a similar mechanism to EDTA, resulting in inhibition of the venom enzymes. These results were in accordance with those of Nithitanakool *et al.* [21] who reported that MSKE exhibited a chelating activity with ferrous metal ions, while Melo *et al.* [22] and Soares *et al.* [23] suggested that the plant extracts causing inhibition of these enzymatic activities have compounds that bind to divalent metal ions, which are required for enzymatic activities. The inhibition of both caseinolytic and fibrinogenolytic activities in CR and NK venoms by MSKE may contribute to the prevention of ECM degradation and thereby decrease the diffusion of toxins through the tissues.

It can be seen from Figure 1 and Figure 2 that MSKE and its phenolic principles (GA, MG and PGG) exhibited concentration−dependent inhibitory effects on caseinolytic and fibrinogenolytic activities of both venoms. These results indicated that the anti−enzymatic potency of MSKE may be attributed to its major principle (PGG) and other unidentified constituents, since PGG exerted its effect at the lowest EC_50_ value and had the highest percentage content (61.28%) within the MSKE compared with GA (0.44%) and MG (0.68%).

### 2.3. Molecular modeling

#### 2.3.1. Docking into the rhodostoxin structure

**Figure 3:**
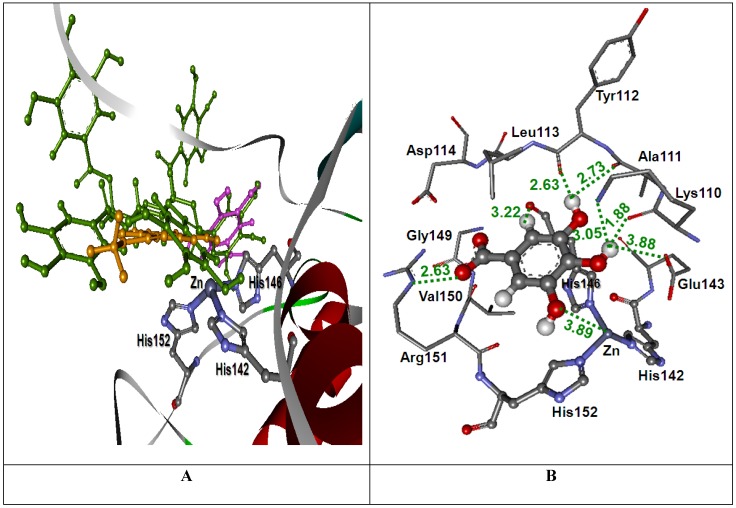

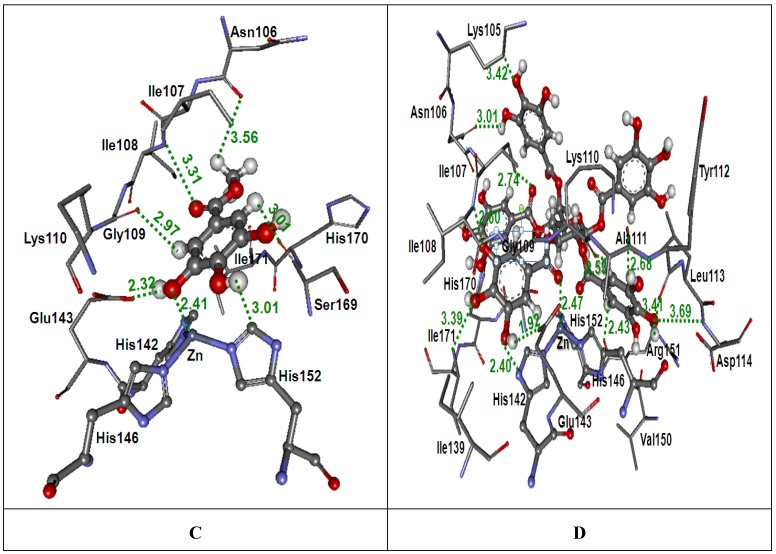
(A) Docked conformation of ligand structures in the binding site of rhodostoxin (GA: Pink, MG: Orange and PGG: Green). (B–D) Distances (in Å) between residues in the rhodostoxin binding pocket and ligands: GA (B), MG (C) and PGG (D).

The results of molecular modeling using a molecular docking method revealed the possible molecular orientation of the MSKE constituents (GA, MG and PGG) in the rhodostoxin (P−I SVMP) binding pocket of CR venom (Figure 3). The GoldScores of GA, MG, and PGG are 47.13, 39.04, and 83.08, respectively (Table 1). The GoldScores revealed that PGG was bound tighter than GA and MG in its binding pocket of CR venom. The binding orientations of GA, MG and PGG in the rhodostoxin are shown in Figure 3A. All docked conformations revealed the strong H−bond with an oxygen atom of Glu143 side chain and another interaction with some residues in the active site. From the docked conformation of GA, H−bond interactions were also found with Lys110, Ala111, Tyr112, His146, and Arg151 (Figure 3B). The closest distance between Zn^2+^ atom and GA’s atom was 3.89 Å, which was the distance between Zn^2+^ atom and O9 atom of GA. In case of MG, the O9 atom of docked conformation showed an interaction with the Zn^2+^ atom with a distance of 2.41 Å. The other interactions were the H−bond interactions formed with Asn106, Ile108, Gly109, His152, and Ser169 (Figure 3C). From Figure 3D, it can be seen that the docked PGG conformation revealed a similar interaction between both docked GA and MG conformations. There were also H−bond interactions with Asn106, Ile108, Gly109, Lys110, Ala111, Tyr112, Arg151, His152, and Ser169. Moreover, H−bond interactions were found with Lys105, Ile107, Leu113, Asp114, His142, Val150, His170, and Ile171. The interaction between the Zn^2+^ atom and the O29 atom of docked PGG conformation was shown to have a distance of 2.49 Å.

#### 2.3.2. Docking into kaouthiagin structure

**Figure 4:**
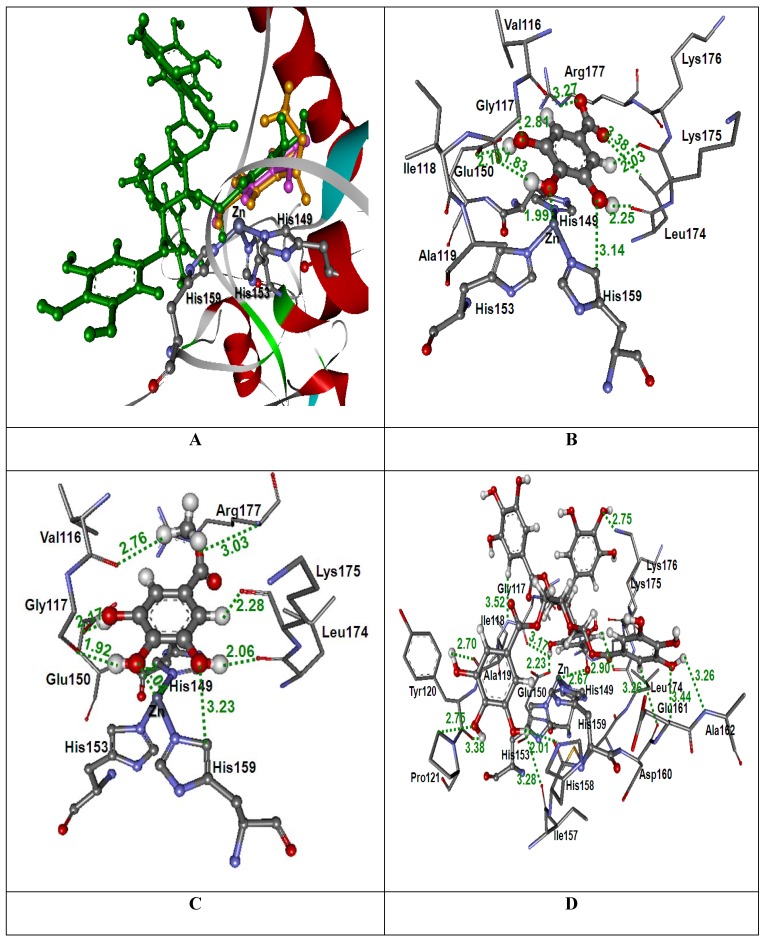
(A) Docked conformation of ligand structures in the binding site of kaouthiagin (GA: Pink, MG: Orange and PGG: Green). (B–D) Distances (in Å) between residues in the kaouthiagin binding pocket and ligands: GA (B), MG (C) and PGG (D).

The docked conformations of GA, MG and PGG in the kaouthiagin (P−III SVMP) binding pocket of NK venom are shown in Figure 4. The GoldScores of GA, MG, and PGG are 47.70, 48.70, and 76.45, respectively (Table 1). The GoldScores revealed that PGG was bound tighter than GA and MG in its binding pocket in NK venom. The binding orientations of GA, MG and PGG in the kaouthiagin are shown in Figure 4A. The docked GA conformation (Figure 4B) was located at a similar position to that of the docked MG conformation (Figure 4C) and the Ring C position of the docked PGG conformation (Figure 4D). The O10 atom of docked GA and MG conformations revealed an interaction with Zn^2+^ atom (1.99 Å and 2.67 Å, respectively) and H−bond interactions were also found with Gly117, His149, Glu150, His159, Leu174, Lys175, and Arg177. A hydrogen atom of a methyl group of the docked MG conformation formed an H−bond interaction with Val116. In the case of docked PGG conformation, the Ring C revealed the same H−bond interaction as shown in the docked GA and MG conformations; i.e. Gly117, His149, Glu150, His159, Leu174, Lys175, and Arg177. The O28 atom of docked PGG conformation was found to interact with Zn^2+^ atom (2.67 Å). The other parts of the docked PGG conformations were formed the H−bond interactions with Val116, Ile118, Ala119, Tyr120, Pro121, Ile157, His158, Asp160, Glu161, Ala162, and Lys176.

It can be seen from Figure 3 and Figure 4 that the tannin principles of MSKE formed hydrogen bonds with the three active histidine residues in the conserved zinc−binding motif (**H**Ebx**H**xbGbx**H**D) of the SVMPs from CR and NK venoms. These active histidine residues are involved in the binding of the catalytically essential zinc ion; if the inhibitors are bonded to these residues the SVMPs catalytic efficiency can be changed [24]. Furthermore, the tannins of MSKE also form important chemical bonds with the Zn^2+^ atom of both SVMPs. Therefore, the results of molecular docking indicated that the phenolic molecules of MSKE could selectively bind to the conserved zinc−binding sites that are critical to the catalysis of SVMPs. In addition, this study found that the phenolic molecules of MSKE can chelate Zn^2+^ atoms and it is well known that SVMPs depend on metal ions (especially Zn^2+^ and Ca^2+^) to exert theirs action. These results were in accordance with those of Nithitanakool *et al.* [21] who found that MSKE was a natural chelator. Since SVMPs have been considered as the key toxins involved in snake venom−induced pathogenesis, example of hemorrhage, edema, hypotension, hypovolemia, inflammation and necrosis [3]; we can confirm from our results the presence of a selective mechanism for MSKE against the activity of SVMPs in CR and NK venoms. In addition, these results supported our previous findings which demonstrated the inhibitory activities of MSKE against hemorrhagic and dermonecrotic activities of CR and NK venoms in mice and rats, respectively [18]. 

## 3. Experimental

### 3.1. Chemicals and venoms

Disodium ethylenediamine tetraacetic acid (EDTA; ≥99.7%) was purchased from J.T. Baker (Phillipsburg, NJ, USA). Gallic acid (GA; ≥98%) and methyl gallate (MG; ≥98%) were purchased from Fluka (Buchs, Switzerland). Pentagalloylglucopyranose (PGG; > 95%) was obtained from Endotherm GmbH (Germany). Casein (≥99%) and type I−S bovine fibrinogen (65−85% protein) were purchased from Sigma Chemical (St. Louis, MO, USA). Lyophilized CR and NK venoms were obtained from the Queen Saovabha Memorial Institute, Thai Red Cross Society, Bangkok, Thailand. Low molecular weight markers (phosphorylase b (97.4 kDa), serum albumin (66.2 kDa), ovalbumin (45 kDa), carbonic anhydrase (31 kDa), trypsin inhibitor (21.5 kDa) and lysozyme (14.4 kDa)) and other chemicals (analytical grade or higher) were obtained from local distributors.

### 3.2. Plant material and extraction

Fully grown unripened Thai mango fruits (*Mangifera indica* L. cv. ‘Fahlun’) were purchased from a local market. A voucher specimen (R.B. 20007) was deposited at the Museum of Natural Medicine, Faculty of Pharmaceutical Sciences, Chulalongkorn University, Bangkok, Thailand. The ethanolic extract from fresh mango seed kernels was obtained by a previously described method [21]. Briefly, the kernels were chopped and homogenized in a blender, using hot ethanol (80 °C) as an extracting solvent at a ratio of seed kernels/solvent = 1:2 (w/v) for 10 min at room temperature (30 °C) prior to centrifugation at 2000 rpm for 15 min in a Hattich Roto magna^®^. The extraction was performed three times. The ethanolic extracts were filtered, combined and concentrated in a rotary evaporator (Büchi Rotavapor R−200) at 40 °C. The extract was defatted with hexane, evaporated under reduced pressure, and then freeze−dried to afford a crude mango seed kernel extract (MSKE) with a yield of 9.36% (w/w).

### 3.3. Standardization

The MSKE was standardized for the contents of GA (0.44%), MG (0.68%) and PGG (61.28%) by using thin layer chromatographic (TLC)/scanning densitometric method [21]. Reference standards of GA, MG and PGG were used as chemical markers.

### 3.4. In vitro test for the inhibition of caseinolytic activity

Caseinolytic activity of CR and NK venoms was measured by modifying the method of Kunitz [25]. One milliliter of 1% casein in 0.1 M Sorensen’s phosphate buffer pH 7.6 and 1 mL of CR or NK venom (10–2500 µg) in the phosphate buffer were incubated for 30 min at 37 °C. The undigested casein was precipitated and the reaction terminated by adding 3 mL of 5% trichloroacetic acid. After centrifugation at 5,000 rpm for 15 min, the absorbance of the supernatant was measured at 280 nm. One unit of caseinolytic activity was defined as an increase of 0.001 absorbance units at 280 nm/h. The initial caseinolytic dose of CR and NK venoms was obtained from the plot between caseinolytic activity and the venom doses. MSKE and its isolated phenolic principles were evaluated for its anti−caseinolytic potential against both venoms. Each 0.5 mL of test solution (with different concentrations) was pre−incubated for 1 h at 37 °C with an equal amount of each venom solution (0.1 mg/mL for CR and 1 mg/mL for NK) before the mixture was subjected to caseinolytic activity evaluation. EDTA was used as a positive reference; blanks containing only the buffer and test sample were run in parallel. The anti−caseinolytic potential of the extract and its phenolic principles was presented as percent inhibition of the enzymatic activity versus concentration. EC_50_, the effective concentration that caseinolytic activity of the venoms was reduced by 50%, was calculated.

### 3.5. In vitro test for the inhibition of fibrinogenolytic activity

Fibrinogenolytic activity of CR and NK venoms was determined by modifying the method of Ouyang and Teng [26]. The reaction mixture (15 μL) contained bovine fibrinogen (16.9 μg) and venom (16.9 μg) in 5 mM Tris−HCl buffer pH 7.4 containing 10 mM NaCl and incubated for 15 min at 37 °C. The incubation was terminated by adding 3.75 μL denaturing buffer 5X containing 0.2 M Tris−HCl (pH 6.8), 20% glycerol, 10% sodium dodecyl sulfate (SDS), 0.05% bromophenol blue and 10 mM β−mercaptoethanol, followed by boiling at 100 °C for 10 min. The samples were analyzed by 15% SDS−PAGE. For the inhibition study, the venom was pre–incubated with different concentrations of the extract or its isolated phenolic principles for 60 min at 37 °C before the mixture was subjected to fibrinogenolytic activity evaluation. EDTA was used as a positive reference; blanks containing only the buffer and test sample were run in parallel.

### 3.6. Molecular modeling

**Figure 5:**
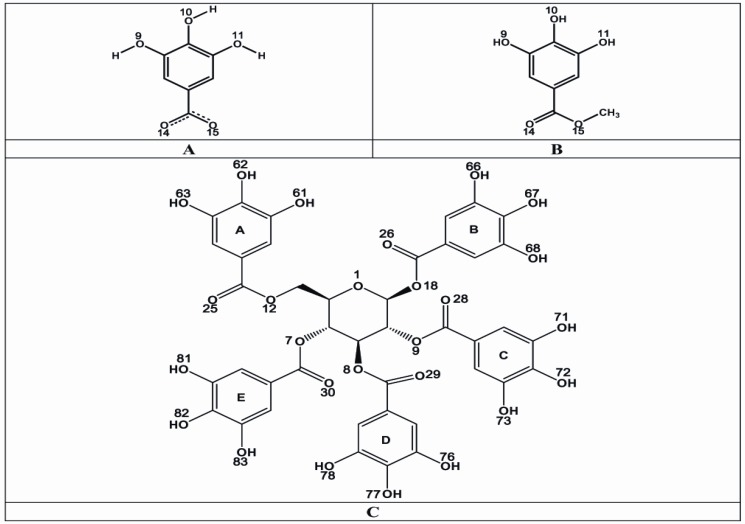
Chemical structures of three major constituents of Thai mango seed kernel extract: GA (A), MG (B) and PGG (C).

Structures of GA, MG and PGG (Figure 5) were constructed and optimized at the HF/3−21G level of theory using Gaussian 03 program [27]. The three−dimensional structures of rhodostoxin and kaouthiagin from CR and NK venoms, respectively, were built by homology modeling technique using Geno3D web server [28].

Geno3D web server used distance geometry, simulated annealing and energy minimization algorithms to build the protein 3D model. After that, the structures were checked for the quality of geometry by using PROCHECK [29]. The rhodostoxin structure was modeled based on the structure of zinc metalloproteinase from the snake venom of *Agkistrodon acutus* (PDB ID: 1BSW) which the % sequence identity was 47. From the PDB ID 1BSW structure, the coordination between Zn^2+^ atom and His142, His146, and His152 residues were shown in the active site. Therefore, the position of Zn^2+^ atom in rhodostoxin structure was set by using the superimposition structure between the rhodostoxin structure and the structure of zinc metalloproteinase from the snake venom of *Agkistrodon acutus*. In case of kaouthiagin, the structure was modeled based on the structure of vascular apoptosis−inducing protein−1 (VAP1) (PDB ID: 2ERP) which the % sequence identity was 54. From the PDB ID 2ERP structure, the coordination between Zn^2+^ atom and His149, His153, and His159 residues were found in the active site, moreover, a GM6001 [(3−(N−hydroxycarboxamido)−2−isobutyl−propanoyl−Trp− methylamide)] inhibitor bound with Zn^2+^ was also found. For the position of metal atoms (Zn^2+^ and Ca^2+^) in kaouthiagin structure, they were set by using the superimposition structure between the kaouthiagin structure and the structure of VAP1. Hydrogen atoms were added to all structures using the SYBYL 7.2 program and the structures were then minimized using Tripos forcefield in the SYBYL 7.2 program (TRIPOS, Assoc., Inc., St. Louis, MO, USA). The molecular docking method was performed using Gold version 4.0 program [30] to investigate the binding orientations of GA, MG and PGG in rhodostoxin from CR venom and kaouthiagin from NK venom. The radius of the binding site was set to 10 Å surrounding the Zn^2+^ atom. The coordination geometry of Zn^2+^ atom was assumed to be tetrahedral geometry. To set the genetic algorithm parameters, the default parameters of automatic settings were used. The GoldScore fitness function was used to determine the fitness score, as shown in GoldScore. The highest GoldScore conformation of each ligand was selected for further analysis.

### 3.7. Statistical analysis

Results were expressed as mean ± S.E.M. for at least two determinations. Statistical analysis was carried out using SPSS 13.0 for Windows. Significant differences (*P* < 0.05) between means were assessed by one−way ANOVA, followed by Tukey’s honesty significant difference test or Dunnett’s T3 test for multiple comparisons.

## 4. Conclusions

MSKE and its major phenolic principle (PGG) possess potent, concentration−dependent anti−caseinolytic and anti−fibrinogenolytic activities against CR and NK venoms. The molecular docking results agreed well with the observed *in vitro* data, in which the anti−caseinolytic and anti-fibrinogenolytic activities of its phenolic principle PGG were higher than those of the other phenolic principles (MG and GA). The docking study revealed the binding orientation of the phenolic principles in the SVMPs binding pocket. The phenolic principles of MSKE formed hydrogen bonds with the three active histidine residues in the conserved zinc−binding motif and could chelate the Zn^2+^ atom of the SVMPs, which resulted in inhibition of the enzymatic activity.

## Figures and Tables

**Table 1:** GoldScores of ligands docked into rhodostoxin of CR and kaouthiagin of NK venoms.

MSKE constituents	GoldScore	
	Rhodostoxin	Kaouthiagin
	(CR venom)	(NK venom)
GA	47.13	47.70
MG	39.04	48.70
PGG	83.08	76.45
